# Canine diabetes mellitus demonstrates multiple markers of chronic inflammation including Th40 cell increases and elevated systemic-immune inflammation index, consistent with autoimmune dysregulation

**DOI:** 10.3389/fimmu.2023.1319947

**Published:** 2024-01-22

**Authors:** Gisela Vaitaitis, Tracy Webb, Craig Webb, Christina Sharkey, Steve Sharkey, Dan Waid, David H. Wagner

**Affiliations:** ^1^ Department of Medicine, The University of Colorado Anschutz Medical Campus, Aurora, CO, United States; ^2^ Department of Clinical Sciences, The Colorado State University Veterinary Teaching Hospital, Fort Collins, CO, United States; ^3^ Department of Clinical Sciences, Montclaire Animal Clinic, Denver, CO, United States; ^4^ Op-T, LLC, Fitzsimmons Innovation Bioscience, Aurora, CO, United States

**Keywords:** canine diabetes mellitus, autoimmune, chronic inflammation, Th40 cells, inflammatory index

## Abstract

**Introduction:**

Canine diabetes mellitus (CDM) is a relatively common endocrine disease in dogs. Many CDM clinical features resemble human type 1 diabetes mellitus (T1DM), but lack of autoimmune biomarkers makes calling the disease autoimmune controversial. Autoimmune biomarkers linking CDM and T1DM would create an alternative model for drug development impacting both human and canine disease.

**Methods:**

We examined peripheral blood of diagnosed CDM dog patients comparing it to healthy control (HC) dogs. Dogs were recruited to a study at the Colorado State University Veterinary Teaching Hospital and blood samples collected for blood chemistry panels, complete blood counts (CBC), and immunologic analysis. Markers of disease progression such as glycated albumin (fructosamine, the canine equivalent of human HbA1c) and c-peptide were addressed.

**Results:**

Significant differences in adaptive immune lymphocytes, innate immune macrophages/monocytes and neutrophils and differences in platelets were detected between CDM and HC based on CBC. Significant differences in serum glucose, cholesterol and the liver function enzyme alkaline phosphatase were also detected. A systemic immune inflammation index (SII) and chronic inflammation index (CII) as measures of dynamic changes in adaptive and innate cells between inflammatory and non-inflammatory conditions were created with highly significant differences between CDM and HC. Th40 cells (CD4+CD40+ T cells) that are demonstrably pathogenic in mouse T1DM and able to differentiate diabetic from non-diabetic subjects in human T1DM were significantly expanded in peripheral blood mononuclear cells.

**Conclusions:**

Based on each clinical finding, CDM can be categorized as an autoimmune condition. The association of significantly elevated Th40 cells in CDM when compared to HC or to osteoarthritis, a chronic but non-autoimmune disease, suggests peripheral blood Th40 cell numbers as a biomarker that reflects CDM chronic inflammation. The differences in SII and CII further underscore those findings.

## Introduction

Canine diabetes mellitus (CDM) is a relatively common endocrine related disease in companion dogs (1). The prevalence ranges from 0.1 to 1.2% accounting for 165,000 diabetic dogs in the U.S., with the incidence rate increasing by 79.7% since 2006 (2) Treatment options are very limited and thus the euthanasia rate is 10% at diagnosis with an additional 10% one year later (2). As with humans, disease etiology is poorly understood. CDM involves persistent hyperglycemia resulting from insulin deficiency caused by massive beta cell loss (3). Symptoms include polyuria, polydipsia, polyphagia, weight loss, cataracts, and chronic or recurring infections as described by the American Veterinary Medical Association. In human subjects, diabetes mellitus (DM) occurs predominantly as type 1 (T1DM) or type 2 (T2DM). T2DM involves insulin resistance while T1DM is classically an autoimmune disease involving both adaptive and innate immune cells that attack the pancreatic islets leading to chronic inflammation defined as immune cell infiltrates in the islets of Langerhans, and subsequent loss of insulin production.

CDM is usually diagnosed in middle and older age adult dogs. In a study of 860 CDM dogs, the mean age of onset was 8.6 years (4). Human T1D is more common in children but the incidence is expanding in young and older adults (5). CDM can occur in any breed, but studies of breed prevalence suggest that there may be some level of genetic predisposition (4). CDM and human DM share multiple comorbidities that include diabetic ketoacidosis, a severe life-threatening metabolic derangement, cataracts, urinary tract infection (UTI), hyper-adrenocorticism, and dermatitis. CDM is a completely insulin dependent condition, but lack of biomarkers make defining it as an immune-mediated disease controversial (6). In human DM autoantibodies have been well described. In CDM autoantibodies (Aab) to insulin, GAD-65 and/or canine islet-antigen-2 (IA-2) have been reported in a small cohort of diabetic dogs (1, 7). Unfortunately, canine autoantibody detection has been notoriously unreliable. Studies showed that GAD65 antibodies were detected in 0 -13% of CD, 0 – 10% tested positive for IA2 antibodies and 0% tested positive for Zinc transporter 8 antibodies (8). A recent study used a nucleic acid programmable protein array platform to query 1700 pancreatic proteins as possible antigens for canine antibodies (9). In that study 6 autoantibodies were identified that had sensitivity greater than 10% (9). Given the difficulty with auto antibody detection in CDM, alternate diagnostic approaches should be developed.

In this report, we examined twelve diabetic companion dogs that were diagnosed at the Colorado State University Veterinary Teaching Hospital (CSU-VTH) in Fort Collins, CO and in a veterinary clinic. Based on history, physical examination, and persistent fasted hyperglycemia and glucosuria, all dogs met the veterinary criteria for CDM. Blood samples were collected for clinical laboratory and research laboratory assessment, analyzed, and compared to healthy control (HC) dogs. The total number of lymphocytes, monocytes, neutrophils, and platelets were examined. We examined serum glucose, cholesterol, and the cholestatic liver enzyme alkaline phosphatase (ALP). Various indices of dynamic leukocyte changes as possible predictors of chronic/systemic inflammation were explored. C-peptide levels as a measure of beta cell function were examined. Levels of glycosylated albumin (i.e., fructosamine) as a measure of chronic hyperglycemia were examined. We discovered that like in human T1DM and laboratory mouse autoimmune diabetes, CDM demonstrates a significant elevation in a subset of peripheral blood cells called Th40 cells (10). The discovery that Th40 cell levels in CDM dogs are substantially and significantly elevated compared to healthy controls supports the use of Th40 cell numbers as a canine biomarker for chronic inflammation. Th40 cells from CDM produce high levels of inflammatory cytokines including IL-6 and IFNγ, consistent with Th40 counterparts from mice (11) and human (12, 13). These findings further underscore that significant increases in Th40 cells correlate with chronic inflammation associated with autoimmunity in multiple species. That, in addition to other findings, supports the claim that CDM is more akin to T1DM, likely latent autoimmune diabetes in adults (LADA), than other forms of DM.

## Materials and methods

### Diabetic dogs

A pilot study to examine chronic inflammatory markers in canine diabetic dogs was conducted at the Colorado State University Veterinary Teaching Hospital (CSU-VTH) in Fort Collins, CO. Dogs from the small animal medicine service or from private veterinary clinics were recruited for the study. An Institutional Review Board (IRB) approval for the pilot study was instituted by the CSU-VTH IRB panel study titled “A Pilot Study comparing Canine Diabetes and Healthy Control Dogs”. At CSU-VTH, dogs that were regular, patients that had been diagnosed with diabetes were recruited; dogs from veterinary clinics were recruited by the attending veterinarians; the same consent process was used. Twelve dogs meeting the criteria for CDM were added to the study. Normal glucose at the CSU-VTH diagnostic lab is 70 – 115 mg/dl blood. Criteria for diagnosis of diabetes was a persistent fasting blood glucose greater than 120 mg/dl and detected glucosuria. Blood and urine for clinical labs, complete blood count (CBC), and research assays were obtained. An Adult Wellness Chemistry panel with SDMA and CBC was performed by a certified clinical laboratory, Antech Diagnostic Laboratory, Englewood CO. Veterinary standard of care was observed; all dogs were prescribed insulin at a dose determined by the attending veterinarian. A description of the dogs is in **Table 1**.

**Table 1:** Description of canine diabetes participants.

*Breed*	*Sex*	*Age in years*	*Time Diabetic in months*
*Terrier mix*	Male	9	6
*Golden*	Female	11	48
*Border collie*	Female	6.2	3
*Yorkshire*	Male	12	1
*Giant Schnauzer*	Female	10	1
*Mix*	Male	8.5	21
*Collie*	Female	13	6
*Cocker spaniel*	Female	9.5	5
*Poodle mix*	Female	11.5	2
*Chihuahua*	Female	8.5	3
*Terrier mix*	Male	6	18
*Boxer mix*	Male	11.5	1

Dogs diagnosed with CDM were recruited. The breeds were mixed in each case. Age and duration of disease is listed.

### Healthy controls

A meta-analysis chart review from a Veterinary Clinic was used for healthy controls (HC). All experiments were approved by the CSU-VTH IRB as described above. Frozen peripheral blood mononuclear cell (PBMC) samples from 11 male and 8 female HC dogs that had undergone spay or neuter surgery or were seen as a healthy checkup, were used. Of those, 7 male and 5 female, had complete records (CBC and Chemistry panel). The dogs were age 9 months – 3 yrs. The breeds were mixed. An Adult Wellness Chemistry with SDMA and CBC was performed by a certified clinical laboratory, Antech Diagnostic Laboratory, Englewood CO. The data were collected from chart review. Permission from owners for access to chart data was obtained by the veterinarians. HC providing research samples were recruited by CSU-VTH veterinarians. These included client-owned dogs seen for wellness checks or minor injuries. Blood and urine samples were processed in the Clinical Pathology laboratory at the VTH. All HC dogs were covered under the approved IRB and all owners signed an approved consent form.

### Research blood samples

Research blood samples, approximately 3 ml, were collected in 10 ml heparinized tubes by venipuncture for analysis. Blood was diluted 1:1 with Phosphate buffered saline (PBS, pH 7.2). Blood was layered over Ficoll-Paque Plus for density gradient separation to collect PBMC. Ficoll-Paque Plus is Ficoll PM400, a copolymerization of sucrose and epichlorohydrin sodium diatrizoate with disodium calcium EDTA. Cells were centrifuged at 1400 rpm (400xg) for 30 min. The buffy coat was removed, washed twice in 10 ml PBS and centrifuged at 300xg to collect cells and remove platelets. PBMC were suspended in a staining buffer, PBS/2 mM EDTA/0.5% bovine serum albumin (BSA) and stained for flow cytometry. Cells were aliquoted to 100,000 cells per well in a 96 well plate. Staining antibodies included: anti-CD3 (FITC) from Bio-Rad, cat# MCA1774F; anti-CD4 (APC) from Bio-Rad, cat#MCA1038APC; anti-CD40 (G28-5; Alexafluor 405) produced in house; there is no canine anti-CD40 antibody available so the G28-5 anti-CD40 that recognizes human CD40 was used. Th40 cells are defined by gating on CD3, then determining the percentage of CD4^+^CD40^+^ cells within the total CD4^+^ population as previously described for human T1D (13, 14).

### Cytokine analysis

Frozen PBMC from nine diabetic dogs were thawed for analysis. Cells were incubated in RPMI culture medium, 5% BSA for 1 hour. Cells were stained for CD3 (FITC); CD4 (PE); CD40 (ViolBlue). Cells were fixed in 2% paraformaldehyde for 10 minutes, then incubated in 100 μl permeabilization buffer (e-Bioscience, San Diego, CA). Cells were treated with biotinylated anti-IL-6 (R&D Systems, a goat polyclonal anti-dog IL-6 IgG) or biotinylated anti-IFNγ (R&D Systems, a goat polyclonal anti-dog IFNγ IgG) for 10 mins. Cells were washed, suspended in permeabilization buffer, and incubated with APC conjugated streptavidin for 10 mins. Cells were washed and analyzed on a Beckman Coulter Cytoflex flow cytometer.

### C-peptide ELISA

C-peptide levels were assayed in plasma (retrieved from the PBMC isolation) using a Canine C-peptide ELISA kit from Sigma Aldrich, cat# EZCCP-47K. The manufacturer protocol was followed. Samples were analyzed no more than one month after acquisition.

### Statistical analysis

Statistical analysis was performed using GraphPad Prism 9.5.1 software. In each case, two-tailed, unpaired t-tests were performed. Statistical significance was confirmed using ANOVA when appropriate.

### Ethics statement

All experiments were carried out in accordance with ARRIVE guidelines. All experiments were performed under a protocol approved by the Colorado State University Veterinary Teaching Hospital IRB. All methods were carried out in accordance with relevant guidelines and regulations.

## Results

### Glucose levels

**Figure 1:**
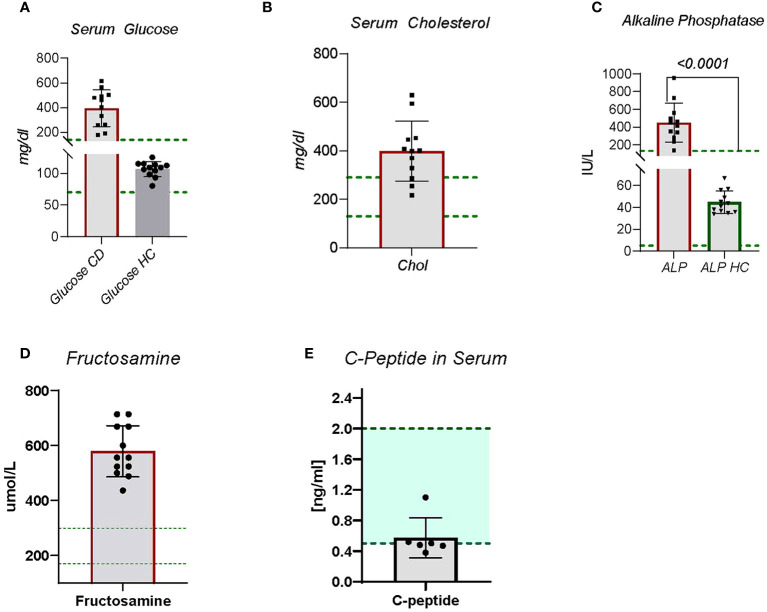
Blood chemistry panels were collected on CDM and HC dogs. Blood was sent to Antech Diagnostic Laboratory for analysis. **(A)** Serum glucose is a standard observation, **(B)** Serum cholesterol is not obtained on most dogs but is included in diabetic dogs due to risk of cardiovascular disease. **(C)** Alkaline Phosphatase, **(D)** Fructosamine, and **(E)** C-Peptide The green lines indicate established normal range. Statistics were done using GraphPad Prism using a two-tailed, unpaired t-test analysis.

Hyperglycemia and glucosuria are hallmarks for CDM and a requirement for the diagnosis and study entry. The CDM study dogs included nine new onset (diagnosed within –1 to 6 months) and three long-term (diagnosed greater than ≥18 months) diabetic dogs. The age, breed, and disease duration of participants is shown in **Table 1**. All diabetic dogs were greater than 5 years of age, which is typical of CDM (2). Regardless of sex or breed, all dogs demonstrated significantly (p < 0.0001) elevated blood glucose compared to HC (**Figure 1A**). The CDM dogs had a glucose range from 176 to 616 mg/dl, with a mean 394.0 ± 42.90 mg/dl (**Figure 1A**). In human subjects, diabetes is diagnosed with a sustained fasting blood glucose level of > 126 mg/dl and/or HBA1c > 6.5%., OR 2-h PG ≥200 mg/dL (11.1 mmol/L) during OGTT; Or patients with classic DM symptoms with random blood glucose > 200 mg/dl (15). In a laboratory setting, diabetes prone mice are diagnosed when blood glucose levels reach and maintain 250 mg/dl (16). Glucose was detected in the urine of all the diabetic dogs, demonstrating glucosuria, which occurs when the blood glucose concentration exceeds the renal threshold for kidney regulation; approximately 180 mg/dL in canines.

### Diabetic ketoacidosis

A criterion for inclusion was glucosuria. Unregulated diabetes can progress to diabetic ketoacidosis (DKA) in both canine (17) and human subjects (18). DKA clinical conditions include blood glucose greater than 250 mg/dl, the presence of ketone bodies by urinalysis, and serum bicarbonate levels less than 18 mmol/L, suggesting acidic conditions (18). Of the 12 dogs in the study, four presented with likely DKA (**Table 2**). They had the highest blood glucose levels, each had detectable ketone bodies in urine, each had a urine pH under physiologic 7.0 indicating acidity, and each had bicarbonate levels under 18 (**Table 2**). Three other dogs were at risk, with a urine pH under 7 and urine glucose ≥ 2.0. Because CDM often goes undetected for long periods of time, DKA development can be common.

**Table 2:** Urine glucose levels. Urine glucose was determined using standard test strips.

Canine Diabetes Urinalysis					
Subject/Age	Spec. Grav.	Protein	Glucose	Ketone	pH
**M, 6 yrs**	1.048	1+	3+	1+	6.0
**F, 11 yrs**	1.013	0	3+	0	8.0
**F, 11.5 yrs**	1.038	1+	1+	0	8.0
**F, 6.2 yrs**	1.038	2+	4+	2+	6.8
**M, 12 yrs**	1.044	0	4+	0	7.0
**F, 9.5 yrs**	1.040	1+	2+	0	6.0
**M, 8.5**	1.031	2+	3+	0	6.5
**F, 13**	1.046	3+	3+	0	5.5
**F, 10**	1.031	2+	3+	1+	5.0
**M, 11.5**	1.035	0	1+	1+	5.8

The range is 0 = no glucose; 1 = up to 100 mg/dl; 2 = up to 250 mg/dl; 3 = up to 500 mg/dl and 4 = > 500 mg/dl. The test strip is also used to measure pH, bilirubin, ketones proteins and blood. The ketone score is neg, 1 = 0.5 mmol/L; 2 = 1.5 mmol/L; 3 = 3.9 mmol/L and 4 up to 10 mmol/L.

### Hypercholesterolemia

During inflammation the increased demand for adaptive and innate immune cells causes increased metabolic requirements. A common mechanism to satisfy metabolic demands is increased cholesterol production and metabolism (19). Cholesterol metabolism plays a key role in maintenance of innate and adaptive immune cells (19). Nine of the twelve CDM dogs demonstrated moderate to severe hypercholesterolemia (**Figure 1B**). Two of the dogs had normal cholesterol levels and the other was borderline high (**Figure 1B**). No direct correlation between serum glucose levels and cholesterol levels were observed, however.

### Alkaline phosphatase

Alkaline phosphatase (ALP) is a zinc modulated enzyme involved in protein metabolism. Increases in ALP often are associated with cholestatic disease, cancers, liver problems, bone disorders, or reactive hepatopathies. In human diabetic subjects increases in ALP have been reported (20). In fact, clinically elevated ALP is associated with increased risk for cardiovascular disease and mortality (20), and for changes in bone density (21). In CDM dogs, ALP was significantly (p < 0.0001) increased compared to HC dogs (**Figure 1C**). All CDM were substantially outside the normal range (**Figure 1C**).

### Fructosamine

A measure of disease control in human diabetes is glycated hemoglobin A1c (Hb A1c). Because of the rapid turnover rate for HbA1C, 14 days compared to 35 days for fructosamine (22), in veterinary patients fructosamine, glycosylated albumin, is examined (23, 24). Fructosamine levels in all diabetic dogs were substantially above the canine normal range (**Figure 1D**), which is consistent with unregulated disease.

### C-peptide

A measure of beta cell function is detection of C-peptide, which is generated when pro-insulin is processed in the islet beta cell to mature insulin. The process creates the mature insulin *A- B* chain molecule and releases C-peptide in serum. When beta cell function is fully dysregulated, C-peptide can no longer be detected. The CDM dogs each had detectable C- peptide, but at levels below the established normal range (**Figure 1E**). This suggests some active beta cells are present in these dogs, but the activity is not sufficient to regulate the blood glucose concentration.

### Lymphocyte dysregulation

**Figure 2:**
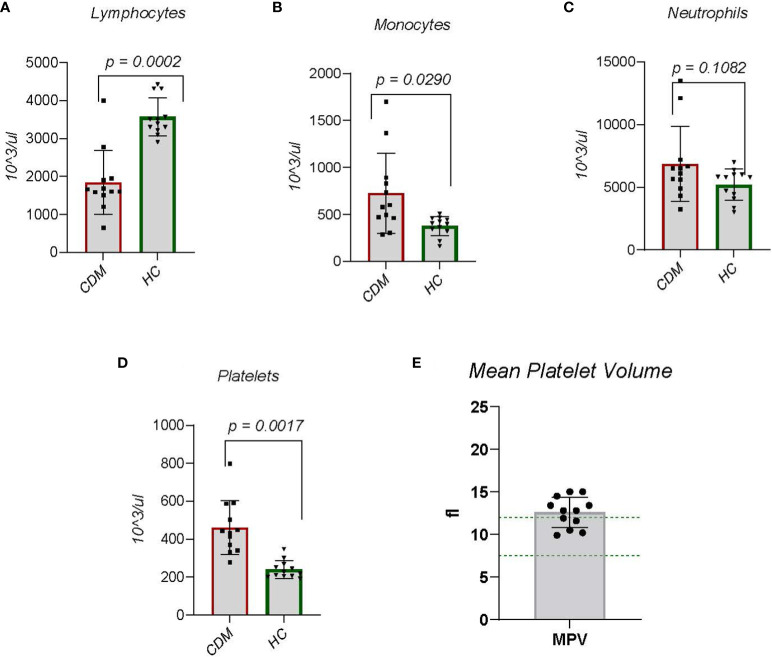
Data are taken from complete blood counts done on each dog; the absolute number per microliter is reported. **(A)** Lymphocytes, **(B)** Monocytes, **(C)** Neutrophils, **(D)** Platelets and **(E)** mean platelet volume (MPV) were done. MPVs were done only on the CDM dogs. Like serum cholesterol, MPV is a special request in the CBC. Statistics were done using GraphPad Prism using a two-tailed, unpaired t-test analysis.

Autoimmune disease necessarily involves immunocyte dysfunction, but in CDM the question arises as to whether the disease etiology is autoimmune. Focusing on complete blood counts from patients, we examined total lymphocyte counts, and innate cells including monocytes and neutrophils. In diabetic subjects, lymphocyte numbers were significantly (p < 0.001) lower than in HC dogs (**Figure 2A**). This observation includes both CD4 and CD8 T cells and B cells as cell type differentiation was not performed; clinical laboratories do not perform this differentiation on canine blood. A decrease in lymphocytes likely relates to changes in metabolism, to be discussed further.

### Innate immune dysregulation

Monocyte numbers representing innate immune cells in CDM dogs were increased significantly (p = 0.0166) compared to HC dogs (**Figure 2B**). Neutrophils, another innate immune cell, produce high levels of NADPH oxidase (NOX-2) which is implicated in autoimmunity and hydrogen peroxide generated from neutrophils suppresses lymphocyte activation and cell numbers (25). While the overall number of neutrophils in CDM was increased there was no significant difference between CDM and HC neutrophil counts (**Figure 2C**). Interestingly, the two CDM dogs that had substantially high neutrophil numbers were new onset diabetics.

### Platelet dysregulation

Platelets play a clear role in hemostasis and thrombosis, but growing evidence suggest a contributory role to chronic inflammation (26, 27). Platelets are derived from megakaryocytes and inflammatory cytokines induce rupture of megakaryocytes to increase platelet numbers (28). In both new onset and long-term CDM, platelet numbers were significantly (p = 0.0007) elevated compared to HC (**Figure 2D**). A feature of platelets that reflects both age of the platelet and platelet activity, i.e. more activated, is platelet size registered by mean platelet volume (MPV) (28). Mean platelet volume in 7 of the 12 CDM dogs was above normal range (**Figure 2E**). MPV from HC was not available.

### Systemic/chronic inflammation index

**Figure 3:**
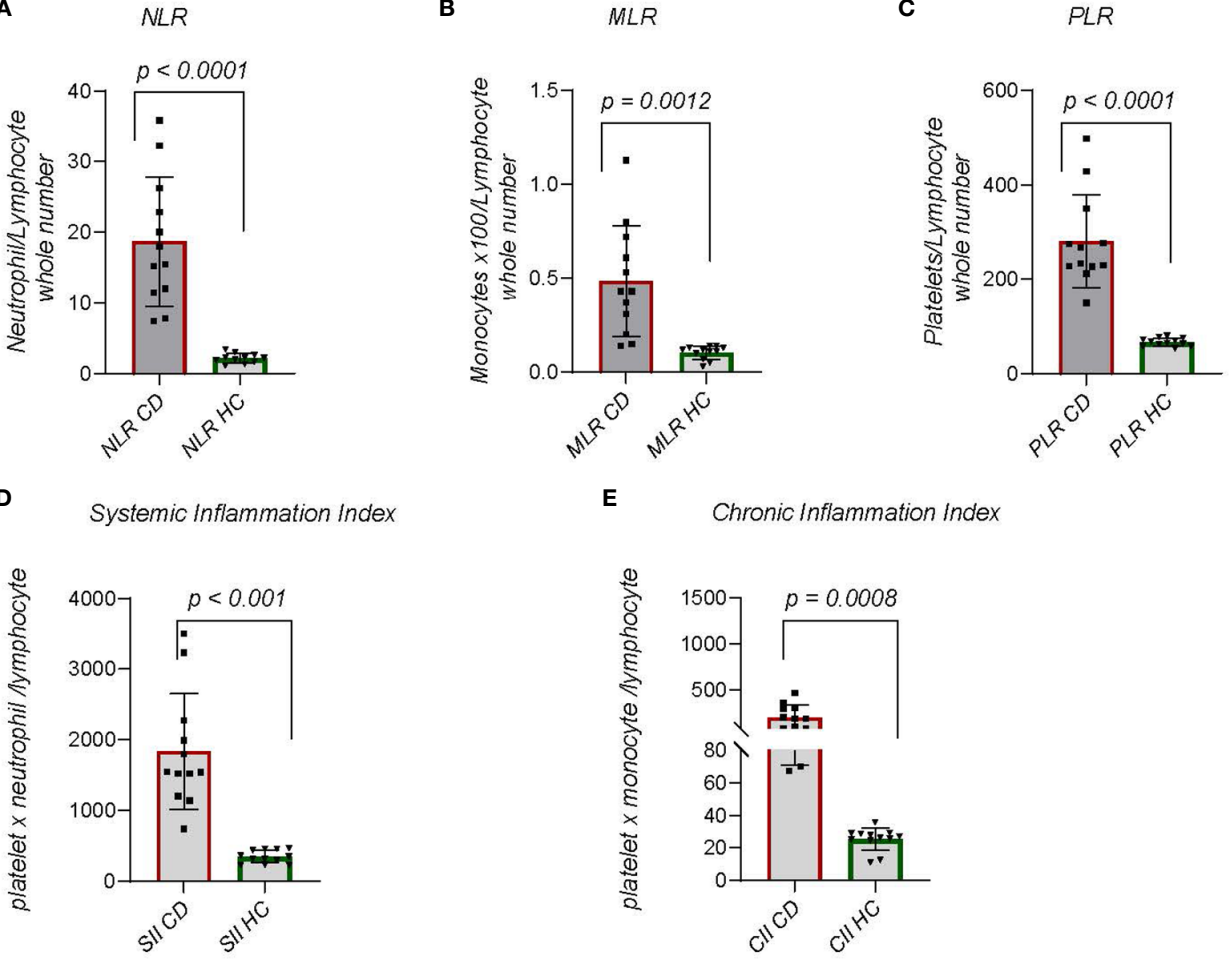
Indices of Chronic Inflammation: **(A)** Neutrophil to lymphocyte ratio (NLR) is calculated by dividing the absolute number of neutrophils per microliter by the absolute number of lymphocytes per microliter of blood. **(B)** Monocyte to lymphocyte ratio (MLR) is the absolute number of monocytes divided by the absolute number of lymphocytes. **(C)** Platelet to lymphocyte (PLR) is the total number of platelets per microliter divided by 10^3^ times absolute number of lymphocytes. (Platelets are reported as whole number x 10^3 per microliter). **(D)** Systemic Immune Inflammation Index (SII) is calculated by (total number of neutrophils) x (whole number of platelets/10^3)/whole number of lymphocytes. **(E)** Chronic Immune Inflammation Index **(CII)** is calculated by: (whole number of monocytes) x (whole number of platelets/10^3)/whole number of lymphocytes. Statistics were done from GraphPad Prism using a two-tailed, unpaired t-test analysis.

Inflammation involves interactions between adaptive immune cells, and innate cells. Attempting to develop inflammatory biomarkers, an index of neutrophil to lymphocyte ratio (NLR) has been created (29, 30). The ratio accounts for dynamic changes in neutrophils and lymphocytes during chronic inflammation. NLR has been described in chronic diseases including coronary heart disease, stroke, diabetes, and cancer of solid organs (30). In one study NLR increases were significantly associated with early neurological deterioration differentiating diabetic and non-diabetic subjects (29). We compared NLR between CDM and HC dogs (**Figure 3A**) with significantly (p < 0.0001) striking differences. All CDM dogs had substantially greater NLR. Another approach focusing on innate to adaptive immune cell differences compared the monocyte to lymphocyte ratio (MLR). MLR is more generally associated with cancers, but has been examined in autoimmune disorders including cardiovascular disease (31). In CDM the MLR was significantly (p = 0.013) greater than in HC dogs (**Figure 3B**). Given the noted differences in platelets between CDM and HC we determined platelet to lymphocyte ratio (PLR). In CDM the PLR was significantly (p < 0.0001) higher than the value in HC dogs (**Figure 3C**).

Further study of dynamic changes in immune cells as a measure of chronic inflammation led to the creation of the systemic-immune-inflammation index (SII), which is derived by multiplying the total number of neutrophils and platelets then dividing by the total number of lymphocytes (32, 33). This index is used for prognosis of solid tumors but also to predict autoimmune outcomes, predominantly heart disease and rheumatoid arthritis (33). In CDM the SII was significantly (p < 0.0001) greater than that in HC dogs (**Figure 3D**). We considered that another analysis could be achieved by substituting monocytes for neutrophils in the index. A chronic inflammation index (CII) was derived by multiplying absolute number of monocytes and platelets then dividing by absolute number of lymphocytes (**Figure 3E**). The difference between CDM and HC dogs was significant (p = 0.0008) and demonstrated a narrower range than the SII.

### Th40 cells indicate chronic inflammation

**Figure 4:**
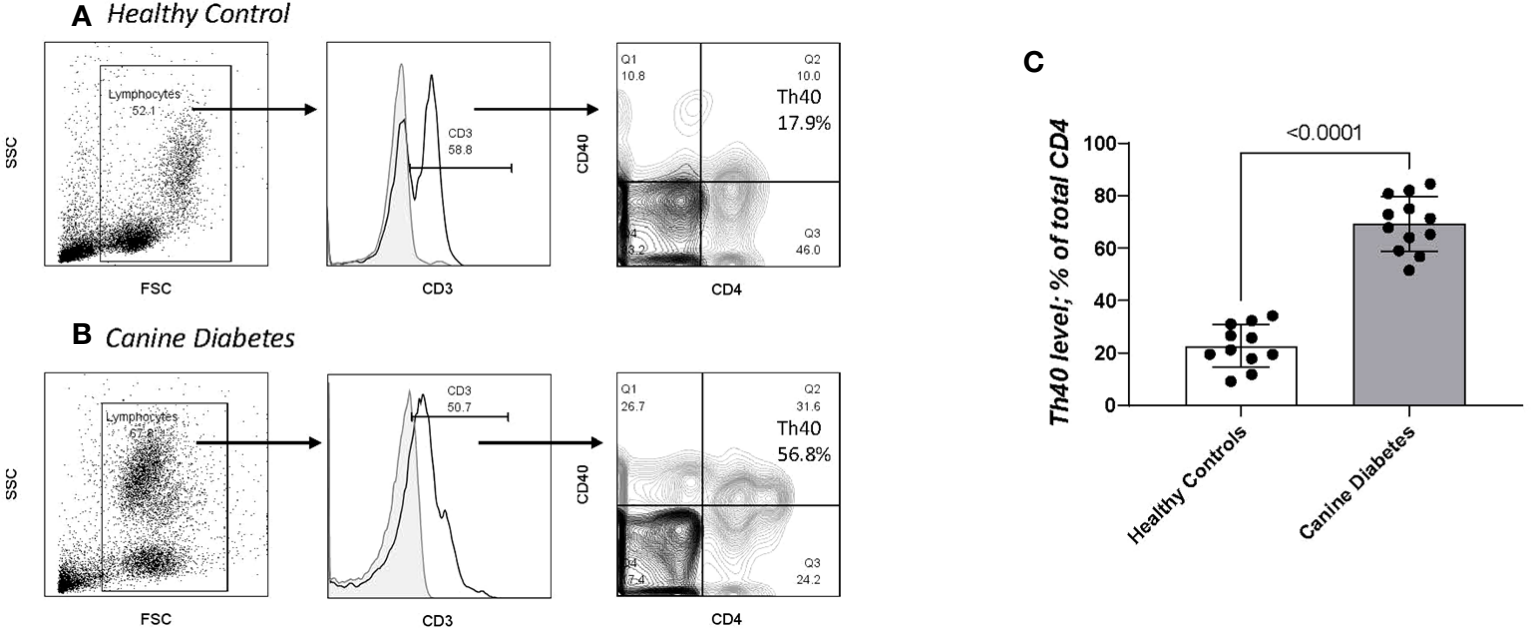
Th40 cell comparison in CD and HC. PBMC analyzed for CD3, CD4 and CD40 (Th40 cells). **(A)** Healthy controls and **(B)** Canine Diabetes were analyzed by dot plots to establish forward versus side scatter. Within the FSC x SSC population CD3+ cells are determined above background controls (grey solid histogram). Within the gated CD3+ population, contour plots of CD4 versus CD40 were determined. Th40 cells are defined as CD3+ then CD4+CD40+/total CD4+ cells (Q2/Q2 + Q3). **(C)** Th40 percentage in CD dogs compared to HC dogs. Statistics were done using Graph Pad Prism using a two-tailed, unpaired t-test analysis.

In previous work we showed that a T cell subset described as Th40; CD3^+^CD4^+^ helper T cells that express the CD40 receptor (10), is increased in number in murine autoimmune diabetes (16, 34, 35) and in human autoimmune (type 1) diabetes (12, 14). In the murine T1DM model, Th40 cells proved to be pathogenic in adoptive transfer studies (16, 34). In human T1DM Th40 cells were present in healthy controls and in type 2 (non-autoimmune) diabetes but at significantly lower numbers compared to T1DM (14). We compared Th40 cell numbers in CDM to HC dogs. Three of the HC dogs were seen for osteoarthritis, a chronic but not autoimmune condition, otherwise they were seen for a healthy dog check-up, vaccines, or elective surgery (spay and neuter). As reported for laboratory mice and human diabetic subjects, CDM dogs had significantly (p < 0.0001) increased Th40 cell numbers (**Figure 4A**). Unlike the reports for variable and highly inconsistent levels of autoantibodies in CDM (3, 9) Th40 cells were universally increased in CDM. PBMC were analyzed for CD3 and then CD4^+^CD40^+^ within the CD3 gated subset (**Figure 4A** for controls and 4B for CDM). In the forward versus side scatter plots, CDM demonstrated a high side scatter, lower forward scatter population (**Figure 4B**) compared to HC dogs (**Figure 4A**). In CDM the CD3 expression was lower than in HC. HC were further examined by age for Th40 cell differences, but no differences were noted (**Supplementary Figure 1A**). There were no differences in Th40 cell numbers when differentiated by sex (**Supplementary Figure 1B)**.

**Figure 5:**
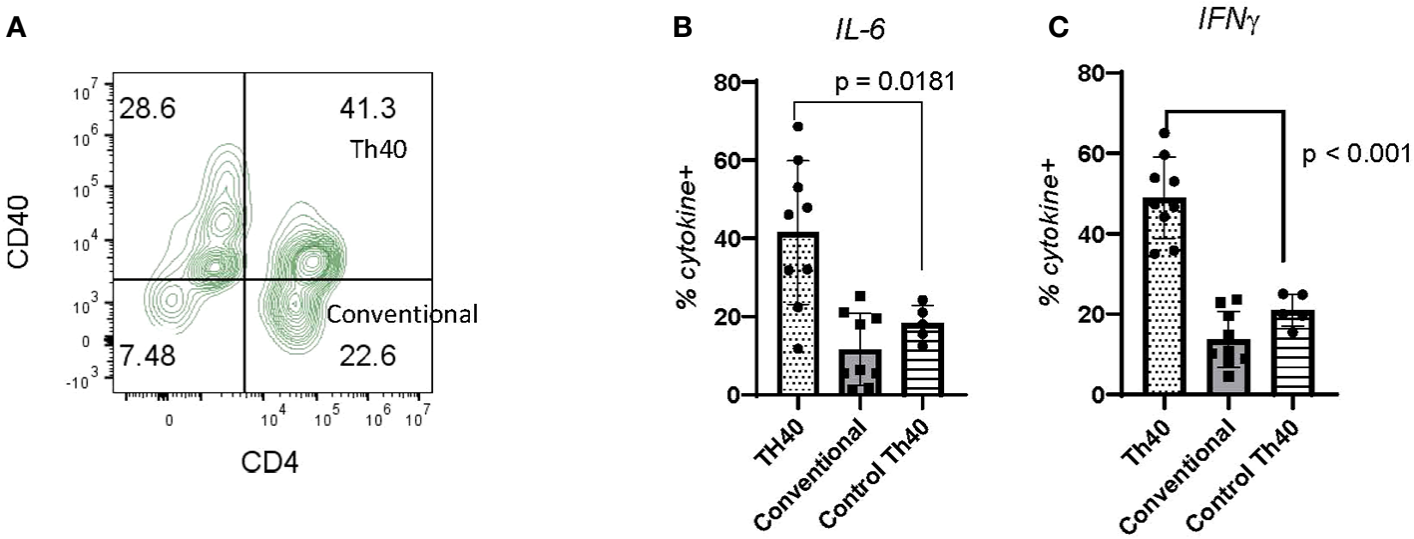
Inflammatory cytokine levels in CDM Th40 cells. PBMC from nine CDM and five healthy controls were analyzed. **(A)** Cells were stained for CD3 then gated against CD4 and CD40; dot plot is representative of CDM. Within the Th40 (CD4+CD40+) and Conventional T cell (CD4+CD40-) populations, intracellular levels of **(B)** IL-6 and **(C)** IFNγ were determined. Statistics were done from GraphPad Prism using a two-tailed, unpaired t-test analysis.

Pathogenicity of CDM Th40 cells: Pathogenicity of Th40 cells was addressed by examining intracellular cytokine levels (**Figure 5**). Th40 cells were gated on CD3 then CD4 versus CD40 was determined (**Figure 5A**) as in **Figure 4**. Levels of intracellular cytokines from CDM dogs were compared to HC for IL-6 (**Figure 5B**) and IFNγ (**Figure 5C**). Th40 versus conventional CD4+ T cells were compared in CDM. For both IL-6 and IFNγ levels in Th40 cells from CDM were significantly higher than levels in HC (**Figures 5B, C**). Likewise, cytokine levels in Th40 were significantly increased compared to levels in conventional cells from CDM (**Figures 5B, C**). While cytokine levels in Th40 cells from HC were significantly less than from CDM, they were increased, although not significant, when compared to conventional cells from CDM.

## Discussion

While CDM has numerous clinical features in common with human type 1 diabetes mellitus, there are also differences (3). CDM is insulin dependent but onset is restricted to mid to late adulthood (1). Human T1D age of onset varies with the peak occurring between ages of 10 -14, mid juvenile (36). An exception is latent adult diabetes autoimmune (LADA) where the average age of onset is 45.02 yrs (37). In CDM, histopathological studies show that beta cell mass is significantly reduced (38) but the cause remains ill defined. It is certainly possible that autoimmune destruction is responsible. In human subjects the presence of autoantibodies (Aab) has been suggested to be predictive for T1DM with some limitations (39). In CDM however, autoantibodies have been disappointingly inconsistent. Reports are that GAD-65 and Islet Antigen- 2 (IA-2) antibodies can be detected, but only in a small population (40). Another study found that antibodies to pro-insulin were detected in 3 of 15 control, and 8 of 15 newly diagnosed diabetic dogs (41). In human disease, GAD and IA-2 auto antibodies are associated with LADA (37). An exploration of Aab comparing 30 healthy controls and 30 CDM dogs found that at a specificity of 90%, six autoantibodies had sensitivity greater than 10% (9). To attempt to address the lack of Aab in CDM a novel array technique that queried 1700 pancreatic proteins was only able to identify 6 autoantibodies with sensitivity greater than 10% (9). Given the reports that autoantibodies are difficult to detect and not proving predictive in CDM, we considered that alternate biomarkers would be beneficial. To that end, we chose to confirm diabetes associated markers, fructosamine, long-standing hyperglycemia and Th40 cells that were shown to be prominent biomarkers in murine (42–45) and human (12–14) T1DM including utility for predicting T1DM onset (12). Th40 cells were in fact more predictive than auto antibodies in human a pre-T1DM study (12).

Islet architecture may contribute to discrepancies between human and canine diabetes. Canine islets average 78% beta cells with cells distributed throughout the islet but concentrated at the center (46). In human pancreas, beta cells are distributed evenly throughout the islet (47). Mice islet architecture is closer to canine with beta cells localized in the center of the islet (3). During murine insulitis (immune cell infiltration of the islet) immune cells invade the poles of the islet, called peri-insulitis, then move to the center to completely engulf the islet with up to 80% of islets demonstrating infiltration (16, 35). Insulitis is rarer in both human and canine diabetes where typically only portions of the islet are infiltrated (3). For example, humans typically have less than 10% of islets infiltrated compared to NOD mice where typically 85% of islets are infiltrated. In CDM the number of immune cells that infiltrate the islet is substantially lower as well (48). In both human and canine diabetes, immune cells likely remain contributory, but not necessarily in the islet.

Another complicating issue is that diabetic dogs demonstrate islet architecture that is substantially different from normal dogs. The CDM islet has only about 30% beta cells compared to 78% beta cells in HC dogs and the islets are dispersed and difficult to identify in CDM (49). Additionally, in CDM, beta cells could be detected in acinar tissue. This leaves the question of whether beta cells were lost due to autoimmune attack, other attack, or the diabetic condition, e.g., canine genetic state causes fewer beta cells. Given that diabetes onsets so late in canines, mid to late adulthood, it is unlikely that the islet architectural differences are genetic, but rather likely occur due to some persistent stimulus, like chronic inflammation. Also, that CDM occurs in a wide range of breeds, argues against genetics as sole contributor.

Assessment of immune contributors in diabetes and particularly in CDM will require focus on circulating immune cells. Here we report that statistically significant differences in peripheral blood cell numbers occur in CDM compared to HC dogs. Monocytes (innate immune cells) were significantly increased while neutrophils trended toward increase but were not statistically different from HC. Monocytes are among the first cells to attack islets where they produce high levels of inflammatory cytokines (50). Monocytes were detected in human islets in post-mortem sections of diabetics both at time of disease (51) and later (52). Neutrophils likewise infiltrate the pancreas (53) to create neutrophil extracellular traps (NETs) which are inflammatory mediators. NETs are a network of extracellular DNA studded with histone and neutrophil granule proteins that trap and damage microbes (54). In addition, NETs create localized inflammation, impacting the inflammasome in autoimmune diseases (55). NETs are increased in serum of human T1D patients compared to healthy subjects (53).

Our observation of decrease in lymphocyte numbers initially was surprising. T cell attack of islets is well established in autoimmune diabetes. In this study and others, decreases in lymphocytes were described based on analysis of CD4^+^CD3^+^ or CD8^+^CD3^+^ T cells and CD19^+^CD20^+^ B cells done by automated clinical laboratory reports. That system does not consider T cell subsets such as Th40 cells. During autoimmune activation classic CD4 and CD8 cells begin to express CD40 (12, 14, 16). Those cells achieve an activated status and CD4 and CD3 cell surface molecules decrease (56). Th40 cells are increased during autoimmune diabetes in mice (16, 34, 35), humans (12, 14) and dogs as shown here. Th40 cells express lower CD4, TCRαβ and CD3 molecules on the cell surface, but in murine studies those molecules had become internalized (57). Thus, the low lymphocyte number seen in CDM could be illusory, in other words, the cells are present but not detected by standard CBC analysis due to the reduction in cell surface CD4, CD8, and CD3 molecules. Another explanation is that innate cell expansion is high energy demanding, lymphocyte numbers may recede due to lack of metabolic expansive energy.

Platelets were described originally for thrombosis, but more recent data show diverse activity including involvement in inflammation. Platelets interact with lymphocytes, monocytes and neutrophils (58) in each case to modulate effector functions. Platelet interaction with lymphocytes increases tissue extravasation, helper differentiation, and cytokine release (58). Platelets express high levels of CD154 (59) and in fact are the primary source of CD154. Platelets from autoimmune subjects express significantly higher levels of CD154 and other inflammatory markers compared to healthy individuals (60). CDM showed significantly increased numbers of platelets, which can drive inflammation associated processes. Platelet derived CD154 interacting with CD40 on monocytes, neutrophils, B cells and Th40 cells would lead to sustained inflammatory cytokine levels. In acute deep thrombosis studies, platelet to lymphocyte (PLR) ratio increase was associated with higher disease risk (32). In rheumatoid arthritis, a chronic systemic autoimmune disease, subjects had significantly higher PLR and NLR compared to healthy controls (61). PLR also differentiates subjects with Hashimoto’s thyroiditis, one of the fastest growing autoimmune diseases in human subjects, from HC (62). Platelets were significantly expanded in CDM as was the PLR. This finding suggests a crucial role for platelets during CDM as well as describing a potential biomarker index for disease diagnosis. High platelet numbers are a source of CD154 and increased Th40 cells are a source of CD40 providing a milieu of increased risk for inflammation development.

Human T1DM is definitionally autoimmune with adaptive immune cells, T and B cells, and innate immune cells including macrophages, dendritic cells, and neutrophils, playing substantive roles in disease development and progression. In early stages, immune cells invade the pancreatic islets (insulitis) focusing attack on the beta cells which leads to a loss of insulin. Human T1DM often includes the presence of autoantibodies (Aabs) (63). Detection of two or more Aabs in children under the age of 5 is considered predictive (39). As subjects age, the presence of Aab and disease predictability reduces substantially (64). We defined another biomarker for chronic inflammation in human T1DM, Th40 cells (12, 65). Th40 cells are CD4^+^ helper T cells that express the CD40 receptor. On these cells CD40 acts as a costimulus driving inflammatory cytokine production whereas CD28, another T cell costimulus, favors regulatory cytokine production (10). Th40 cells in peripheral blood of T1DM subjects were found to be elevated up to 6 standard deviations above healthy controls (12, 14). In a defined at-risk pre-T1DM cohort peripheral blood Th40 cells were also significantly increased in number (12). In peripheral blood of CDM subjects Th40 cell numbers were uniformly expanded in number, compared to HC, including patients examined for osteoarthritis, a chronic but non autoimmune disease. Given the difficulty obtaining differentiated lymphocytes, CD4, CD8, B cell, from standard CBC laboratory tests, this finding offers a direct link to measure chronic inflammation in CDM.

At present, the evidence to fully classify CDM as type 1 diabetes is insufficient. The data presented here demonstrate that CDM carries multiple indicators of chronic inflammation. Another finding is that Th40 cells directly associated with human and mouse autoimmune diabetes is significantly elevated in CDM. Human and murine T1D have definitive characteristics, immune cell infiltrations, autoantibody production, elevated numbers of Th40 cells, etc. NOD mice were genetically bred to develop T1D, and NOD mice carry a single MHC-II haplotype, I-A^g7^. NOD mice clearly are a homogenous model. While proving highly useful, all the treatments developed from the NOD mouse model have yet to be translated clinically. CDM appears to be more like human T1D, perhaps more like LADA, based on islet architecture and other parameters. Generating biomarkers for chronic inflammation during diabetes is crucial to better understanding the disease. Such understanding is critical for the development of therapeutics to target chronic inflammation and the consequences of diabetes. Further work with this model is needed.

## Data availability statement

The original contributions presented in the study are included in the article/**Supplementary Material**. Further inquiries can be directed to the corresponding author.

## Ethics statement

The animal studies were approved by Colorado State University Veterinary Teaching Hospital. The studies were conducted in accordance with the local legislation and institutional requirements. Written informed consent was obtained from the owners for the participation of their animals in this study.

## Author contributions

GV: Data curation, Investigation, Methodology, Writing – review & editing. TW: Data curation, Investigation, Methodology, Writing – review & editing. CW: Data curation, Investigation, Methodology, Writing – review & editing. CS: Writing – original draft. SS: Writing – original draft. DW: Investigation, Methodology, Supervision, Writing – review & editing. DHW: Conceptualization, Data curation, Formal analysis, Funding acquisition, Investigation, Resources, Supervision, Writing – original draft.
