# Supplementary figures and images for: Canine diabetes mellitus demonstrates multiple markers of chronic inflammation including Th40 cell increases and elevated systemic-immune inflammation index, consistent with autoimmune dysregulation
Source: Front Immunol. 2024 Jan 22;14:1319947. doi: 10.3389/fimmu.2023.1319947 (PMC10839093; doi:10.3389/fimmu.2023.1319947)

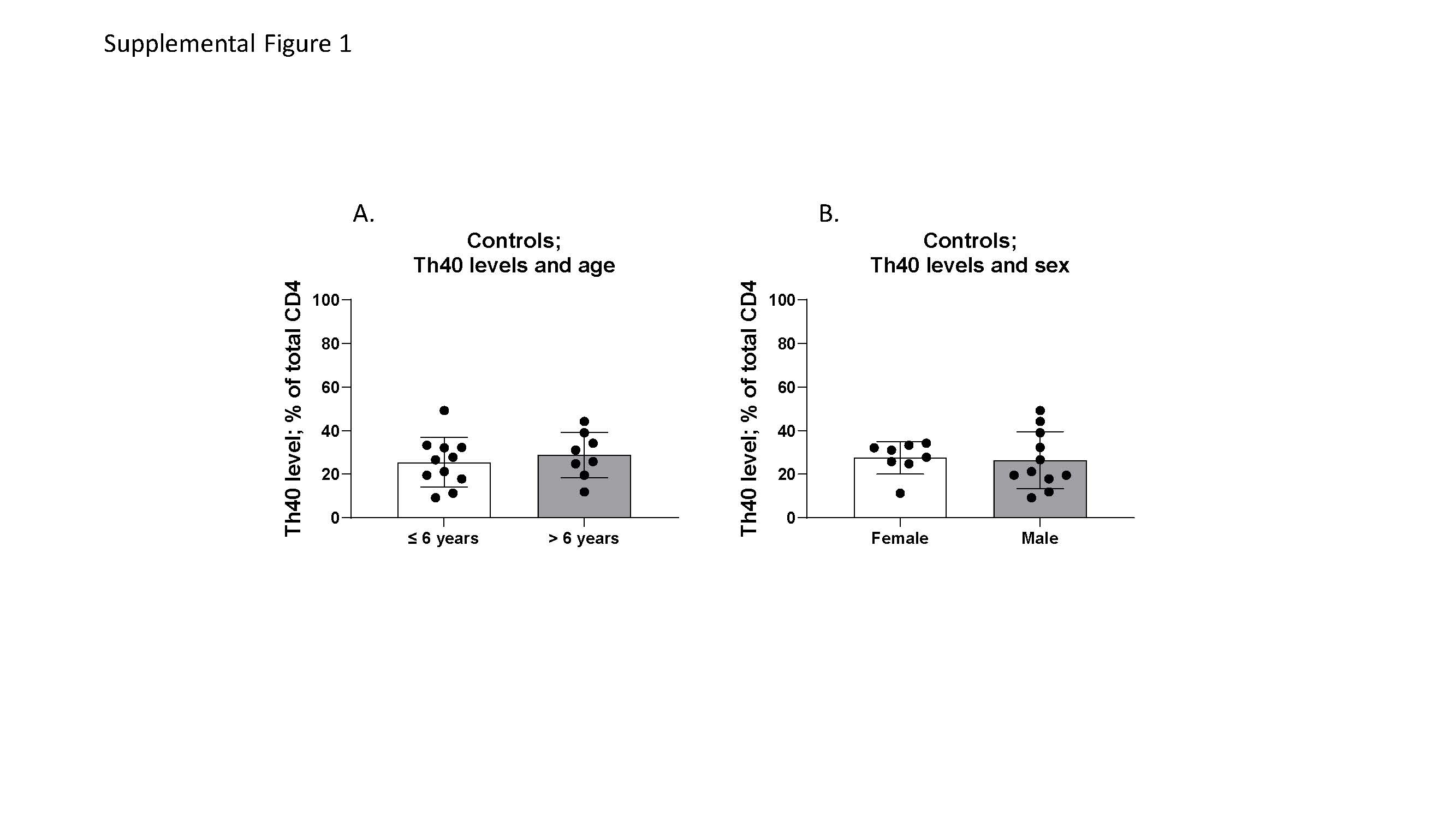

Supplement: Supplementary file 1 [file Image_1.jpg]
